# *Brucella* cervical spondylitis complicated by spinal cord compression: a case report

**DOI:** 10.4076/1757-1626-2-6698

**Published:** 2009-07-09

**Authors:** Paraskevas Hantzidis, Anestis Papadopoulos, Christos Kalabakos, Loukas Boursinos, Christos G Dimitriou

**Affiliations:** Orthopaedic Department, Hippokratio General Hospital of ThessalonikiThessalonikiGreece

## Abstract

A case of 65-year-old farmer who presented with *Brucella*-related cervical spondylitis is described. Because of the advanced form of the infection resulted in neurological impairment, cervical vertebra corpectomy and debridement of the paravertebral granulomatous tissue deposits were performed followed by stabilization with anterior plating and bone grafting. In addition, double antimicrobial chemotherapy regimen was administered for 12 weeks. After one year, follow up evaluation demonstrated resolution of the infection. The authors recommend that brucellosis should be included in the differential diagnosis of cervical spondylitis, particularly in patients who reside in countries where the zoonosis is still endemic.

## Introduction

Brucellosis is caused by aerobic, non-motile, Gram-negative, facultative intracellular, *Capnophilic coccobacilli* belonging to the genus *Brucella*. The genus was named after David Bruce who first isolated this bacteria in 1887 from soldiers with Malta fever [1]. *Brucella* genus has been divided into six species, four of which are known to produce disease in man: *B.abortus*, *B.suis*, *B.canis*, *B.melitensis*, the last being the most virulent. Brucellosis is found primarily in animals and is spread to humans by direct contact with infected tissue or by ingestion of infected animal products, most commonly milk or milk products. In developed countries, human brucellosis is primarily an occupation-related disease while it is known to occur more commonly among farmers, veterinarians and laboratory workers. In developing areas of the world the ingestion of unpasteurized milk represents a common source of infection. Brucellosis exists word-wide presenting higher incidence in the Mediterranean basin, the Arabian peninsula, the Indian subcontinent and South America [2-5]. Osteoarticular involvement including spondylitis, sacroiliitis, osteomyelitis, peripheral arthritis, bursitis and tenosynovitis represents the most common complication of brucellosis affecting up to 85% of patients [6]. The regional distribution of the spondylitis lesions showed a predilection to the lumbar spine (L-4 was most frequently affected) followed by the cervical and thoracic spine [7,8].

## Case presentation

**Figure 1.**
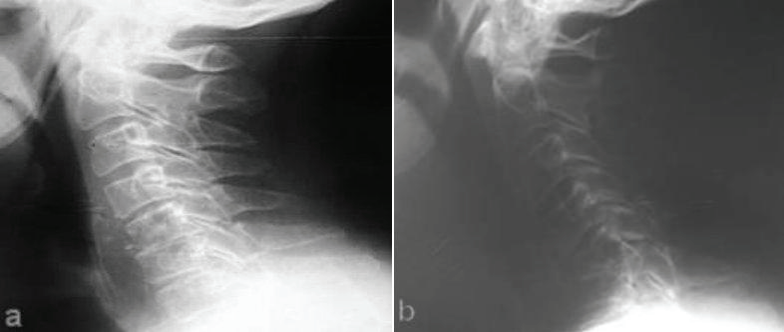
Lateral radiographs of the cervical spine, **(a)** at admission, radiograph shows erosions at the level of the inferior end plate of C5 and superior end plate of C6 vertebra as well as severe narrowing of the intervening disk space, **(b)** after 15 days, dramatic deterioration of the findings is shown while collapse of the C5 vertebra and complete loss of the C5-C6 disk height are observed.

**Figure 2.**
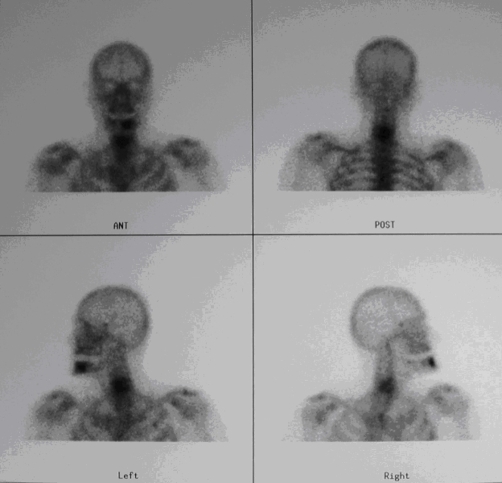
Bone scintigram demonstrates increased uptake in the bodies of C5 and C6 vertebra on all views indicating diffuse form of spondylitis.

**Figure 3.**
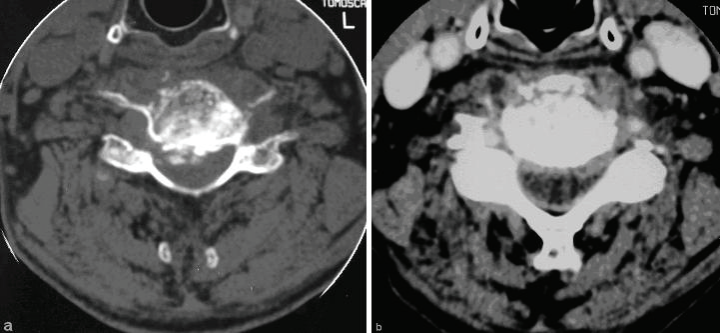
**(a)** Axial CT image at the level of C5 vertebra revealing lytic lesions of the vertebral body, paravertebral granulomatous masses and compression of the spinal cord, **(b)** Axial CT image obtained at the level of the superior end plate of C6 showing erosive changes.

A 65-year-old Greek farmer presented with a 2-month history of fever, progressive malaise and painful neck. No history of trauma was recorded. On admission, neurological examination revealed motor and sensory deficits in distribution of C6 neurotome. Sedimentation rate, C-reactive protein and immunoglobulins Ig A and Ig G values were abnormally high. The patient had positive Wright serum agglutination reaction at titer of 1/160. Plain radiographs of the cervical spine showed bony erosion at the level of C5 and C6 vertebra and collapse of the intervening space (Figure 1). Radionuclide bone scan demonstrated pathological activity on all views of the involved region (Figure 2). CT imaging (Figure 3) demonstrated lytic lesion of C5 vertebra and paravertebral mass at the level of C5-C6 intervertebral space. Also, the spinal cord was compressed significantly and displaced posteriorly. Because of implanted old-fashioned pacemaker MRI evaluation of the cervical spine was contraindicated.

**Figure 4.**
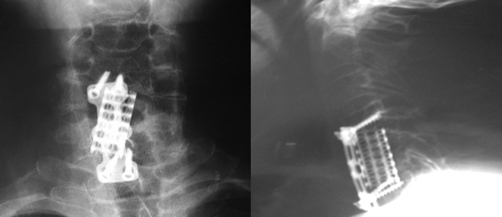
Plain radiographs of the cervical spine obtained at the postoperative evaluation showing the anterior plating and bone grafting (titanium mesh cylinder) of the involved spinal segment.

A right anterior cervical approach was performed. Total corpectomy of C5 and partial corpectomy of C6 vertebra was carried out. Paravertebral mass was dissected from the epidural space and debridement followed. An anterior cervical plate system and bone grafting using titanium mesh cylinder were applied for stabilization (Figure 4).

Biopsy results showed diffuse nonspecific inflammation with granulomatous fibrous deposits. Culture of tissue specimens was negative for the development of fungi or mycobacteria. Postoperatively, improvement of neurological status was observed. He was given 200 mg doxycycline and 100 mg streptomycin daily for 12 weeks. At 1-year follow up, the brucellosis was resolved although the neurological recovery was partial.

## Discussion

Diagnosis of spinal brucellosis is established if the patient presents at least two of the following findings: 1) a blood and/or bone marrow aspirate culture positive for *Brucella*, 2) a *Brucella* agglutination titer of 1:160 or higher, 3) a bone scan, radiographs and/or a computer tomography scan showing skeletal involvement characteristic of osteomyelitis and 4) biopsy suggestive of brucellosis demonstrating noncaseating granulomatous tissue [9].

Two forms of spinal brucellosis have been identified: focal and diffuse [10]. In the focal form, spondylitis is localized to the anterior aspect of a superior end plate, an area that is known for its rich blood supply. The intervertebral disk, paraspinal soft tissues and spinal canal are not affected. If the infection is caused by species with high virulence capacity such as *B.melitensis*, progression to a diffuse form can occur spreading via the ligaments and vascular anastomoses and involving adjacent disks and vertebral bodies. Bone destruction usually results in herniation of disk material into the end plates and spread of the infection into neighboring soft tissues as well as intraspinal extension of granulation tissue with or without cord compression.

Brucellosis should be included in the differential diagnosis of painful and stiff neck in patients who reside in countries where the disease is still endemic or who have traveled to these countries [11,12]. A patient with neck pain with or without neurological deficits, a recent history of low-grade fever and malaise should undergo repeated *Brucella* agglutination tests as a basic part of screening. Also, brucellosis should be included in the differential diagnosis of non-traumatic atlantoaxial rotatory subluxation (Grisel`s syndrome) [13]. Relating with the specific serological findings in patients with brucellosis, positive agglutination to antibodies to *Brucella* bacteria at a dilution of 1:160 or more is usually revealed. It should be kept in mind that sometimes there are false positive results as a result of cross-reacting antibodies during infections caused by *Fransicella tularensis*, *Yersinia enterocolitica* and various *Salmonella* species as well as after vaccination for cholera. Regarding the imaging features of the brucellar cervical spondylitis [1,8,14], plain radiographs are helpful but surely not conclusive. The earliest radiographic signs are bone erosion mimicking sometimes a Schmorl node, bone sclerosis, anterior osteophyte formation in the superior aspect of the affected vertebral body (parrot’s beak) or disk gas (vacuum phenomenon). As the disease advances, destruction of vertebral end plate and narrowing or even collapse of the disc space are observed. As a result of extended lytic lesions, vertebral collapse and spondylolisthesis may be demonstrated. It should be noted that the interval between onset of symptoms of spinal involvement and the appearance of erosive changes on plain radiographs of the cervical spine is about three months. In the absence of changes on plain radiographs, radionuclide bone scans have been shown to be useful demonstrating areas of increased uptake. In the focal form, the scintigraphy usually demonstrates a moderate increase in uptake on the anterior view only. In the diffuse form, increased circumferential uptake is observed. CT imaging can depict not only the vertebral body changes but also any lesion involving the spinal canal and the paravertebral soft tissues. CT is also useful in performing image-guided biopsies of the vertebral body or aspiration of the paravertebral abscess. Unfortunately, computed tomography imaging is invaluable in the differential diagnosis of spinal brucellosis, particularly for tuberculosis of the spine and metastatic tumors. MR imaging is the method of choice for the diagnostic assessment and follow-up of the spinal brucellosis, in particular that of diffuse form which is complicated by lesions involving the spinal canal, nerve roots or paravertebral tissues.

The principal treatment of brucellar spondylitis is conservative, namely, immobilization of the affected area and antimicrobial therapy [15]. An open biopsy of the cervical vertebral body or extradural tissues should be indicated when the differential diagnosis of spondylitis cannot be made by the conventional diagnostic methods. Surgery for decompression of the spinal cord or nerve roots is essential in cases with neurological deficits. Surgical management is also indicated in the presence of poor response to antimicrobial chemotherapy or certain complications such as instability of the affected spinal segment and progressive collapse of vertebral body.

## Abbreviations

CT: computed tomography
Ig: immunoglobulin
mg: milligram
MRI: magnetic resonance imaging
